# The antiangiogenic action of cisplatin on endothelial cells is mediated through the release of tissue inhibitor of matrix metalloproteinases-1 from lung cancer cells

**DOI:** 10.18632/oncotarget.25954

**Published:** 2018-09-25

**Authors:** Robert Ramer, Tilman Schmied, Christin Wagner, Maria Haustein, Burkhard Hinz

**Affiliations:** ^1^ Institute of Pharmacology and Toxicology, Rostock University Medical Center, Rostock, Germany

**Keywords:** cisplatin, low-dose metronomic treatment, tumor angiogenesis, tissue inhibitor of matrix metalloproteinases-1, lung cancer cells

## Abstract

In addition to suppressing cancer cell proliferation and tumor growth, cisplatin has been shown to inhibit tumor angiogenesis. However, the underlying mechanism remains a matter of debate. The present study addressed the impact of cisplatin on potential tumor-to-endothelial cell communication conferring an antiangiogenic effect. For this purpose, migration and tube formation of human umbilical vein endothelial cells (HUVECs) exposed to conditioned media (CM) from vehicle- or cisplatin-treated A549 and H358 lung cancer cells were quantified. Cancer cells were exposed to non-toxic concentrations of cisplatin to mimic low-dose treatment conditions. CM from cancer cells exposed to cisplatin at concentrations of 0.01 to 1 µM elicited a concentration-dependent decrease in HUVEC migration and tube formation as compared with CM from vehicle-treated cells. The viability of HUVECs was virtually unaltered under these conditions. siRNA approaches revealed cisplatin-induced expression and subsequent release of tissue inhibitor of matrix metalloproteinases-1 (TIMP-1) by lung cancer cells to be causally linked to a decrease in HUVEC migration and tube formation. Moreover, TIMP-1 upregulation and consequent inhibition of HUVEC migration by cisplatin was shown to be dependent on activation of p38 and p42/44 mitogen-activated protein kinases. Inhibition of angiogenic features was not observed when HUVECs were directly exposed to cisplatin. Similarly, antiangiogenic effects were not detectable in HUVECs exposed to CM from the cisplatin-challenged bronchial non-cancer cell line BEAS-2B. Collectively, the present data suggest a pivotal role of cisplatin-induced TIMP-1 release from lung cancer cells in tumor-to-endothelial cell communication resulting in a reduced cancer-associated angiogenic impact on endothelial cells.

## INTRODUCTION

Angiogenesis is known as a hallmark of cancer progression for growth of solid tumors beyond 1–2 mm³ [1]. Rapidly growing cancers increase their vascular supply to maintain the delivery of oxygen and nutrition as well as the disposal of cellular waste. Oxygen- and nutrition-starved tumors secrete proangiogenic factors, such as vascular endothelial growth factor (VEGF), which target endothelial receptor tyrosine kinases on the surface of vessel cells to promote neovascularization [2]. At present, antiangiogenic drugs such as the VEGF antibody, bevacizumab, in addition to several small molecule VEGF receptor tyrosine kinase inhibitors (sunitinib, sorafenib, pazopanib) are an integral part of the armamentarium to combat cancer diseases [3].

Platinum-based chemotherapy is the recommended first-line treatment for the majority of advanced inoperable lung cancers [4, 5], with the exception of inhibitors of epidermal growth factor receptor (EGFR) tyrosine kinase as the first-line therapy for patients with an activating EGFR mutation [5, 6]. In particular, cisplatin has been demonstrated to elicit antiangiogenic effects in solid tumors, and has recently been shown to be effective against xenografts of renal cell carcinoma [7], ovarian [8–11], gastric [12], and lung cancer cells [13–15], with the latter including tumors generated by the lung cancer cell line A549, which is also used in the current study [14, 15]. Noteworthy, animal studies performed using A549 cells were not aimed at elucidating the mechanisms of cisplatin-induced antiangiogenesis. Finally, one study further demonstrated the antiangiogenic properties of cisplatin in an *in vivo* alginate-encapsulated ovarian cancer cell assay [10]. However, none of these investigations have addressed a probable cisplatin-modulated tumor-to-endothelial communication conferring antiangiogenesis.

In recent years, low-dose metronomic (LDM) treatment has gained interest as an effective therapeutic option with an improved safety profile [16] that targets tumor neovascularization (for review see [17]). LDM treatment involves the continuous and frequent administration of cisplatin or other chemotherapeutic drugs at doses far below the maximum tolerated doses. Notably, in a study of cisplatin LDM treatment, dosages between 1 mg/m^2^/day and 4 mg/m^2^/day administered 5 days per week yielded the highest serum concentrations on day 26 of the course of approximately 1 and 3 µM cisplatin, respectively [18]. In another study using an LDM dosage regimen of 10 mg/m^2^ twice per week, serum cisplatin concentrations of 0.8, 1.6, and 2.6 µM were measured on day 4, 11, and 25, respectively [19]. Conversely, intravenous bolus injections of cisplatin administered at the maximum tolerated dose of 100 mg/m^2^ elicited total plasma levels of 20.7 µM with unbound intact cisplatin reaching a maximal plasma concentration of 10.9 µM [20].

Data obtained in rodents have highlighted antiangiogenesis induced by LDM treatment with cisplatin as a key mechanism of its tumor-regressive effect on liver cancer [21]. Another investigation showed that LDM treatment with cisplatin reduced vessel density in a xenograft model of head and neck squamous cell carcinoma [22] and inhibited tumor growth via an antiangiogenic action in a murine model of transitional cell carcinoma [23]. The mechanism that confers low-dose cisplatin-induced antiangiogenesis, however, remains unclear. Despite an inhibition of endothelial cell migration and tube formation being shown for other chemotherapeutics including docetaxel, epothilone B, and vinblastine, cisplatin was virtually inactive in this respect [24].

Recently, we have provided evidence that cannabinoids confer tumor-to-endothelial interaction via upregulation of tissue inhibitor of matrix metalloproteinases-1 (TIMP-1) release from lung cancer cells, resulting in a decrease in angiogenic features of human umbilical vein endothelial cells (HUVECs) [25]. Considering that cisplatin has been found to similarly induce TIMP-1 as part of its anti-invasive action on cervical and lung cancer cells [26], the present study addressed a probable TIMP-1-dependent antiangiogenic action of cisplatin at non-toxic concentrations. To this end, a tumor-to-endothelial cell interaction was investigated using the non-small cell lung cancer (NSCLC) cell lines, A549 and H358, according to a recently established protocol [25]. Here, we provide first-time proof for cisplatin-induced TIMP-1 release from lung cancer cell lines to inhibit angiogenic capacities of endothelial cells. These findings may represent a novel antiangiogenic mechanism involved in the antitumorigenic effects of low-dose cisplatin treatment.

## RESULTS

### Impact of cisplatin on lung cancer and bronchial epithelial cell viability

**Figure 1:**
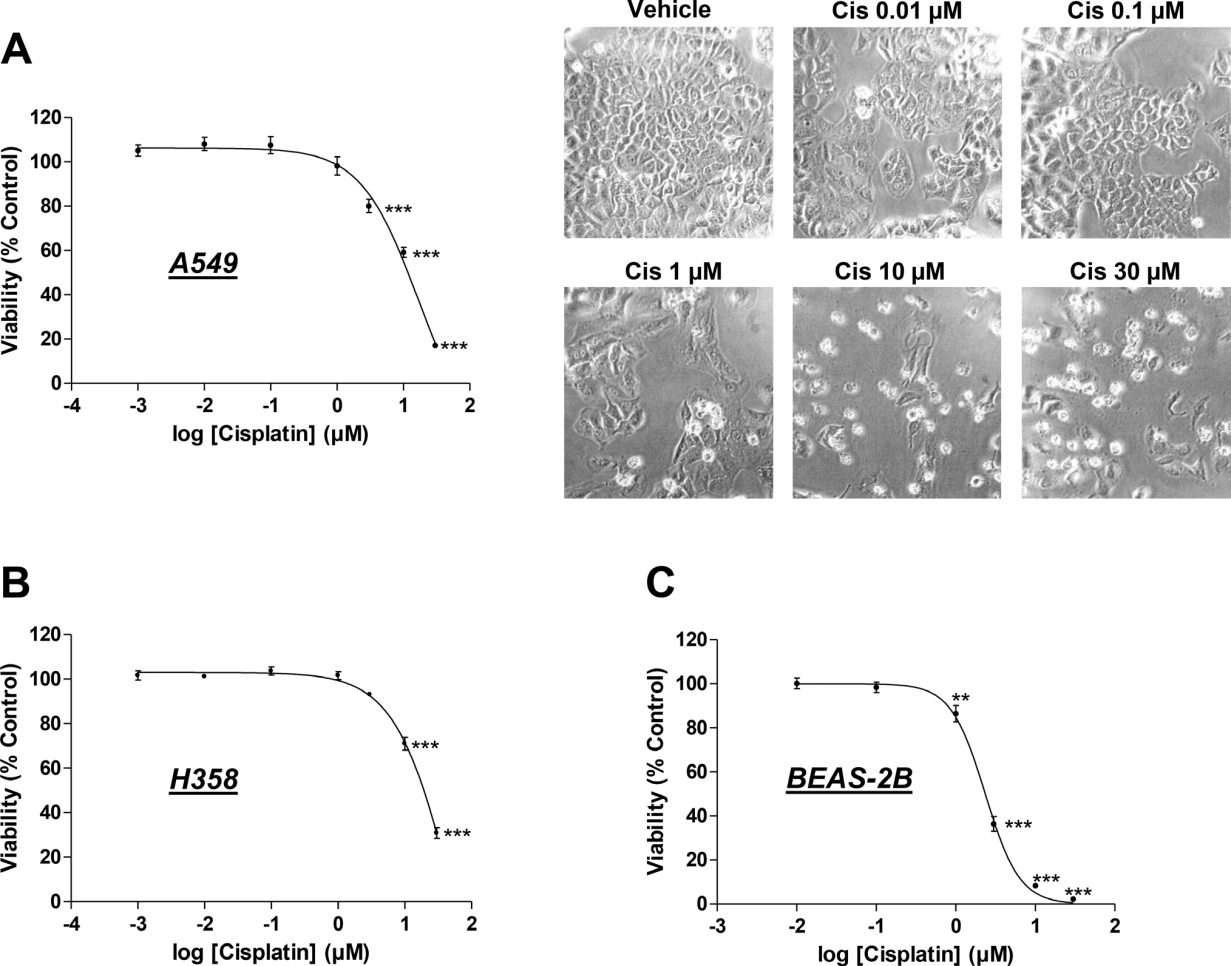
Impact of cisplatin on the viability of the lung cancer cell lines, A549 and H358, and the non-cancer bronchial epithelial cell line, BEAS-2B Viability of A549 (**A**), H358 (**B**), and BEAS-2B (**C**) cells was determined following a 48-h incubation with vehicle or the indicated concentrations of cisplatin in serum-free DMEM using the WST-1 assay. Images at the upper right side depict A549 cells after a 48-h treatment with cisplatin. Percentage of control represents comparison with vehicle-treated cells (set as 100%) in the absence of cisplatin. Values are the mean ± SEM of *n* = 18-20 (**A**, **B**) or *n* = 21 (**C**). ^**^p < 0.01, ^***^p < 0.001 vs. corresponding vehicle control, one-way ANOVA plus a *post-hoc* Dunnett test.

Initial experiments were carried out to monitor the toxicity of cisplatin toward cells used in the present study, with a view to excluding nonspecific toxic effects in the subsequent experiments that would assess its impact on angiogenesis. Accordingly, to provide conditions that maintain the impact of cisplatin on lung cancer cells within a non-toxic range, initial experiments were performed to determine non-toxic concentrations of the drug within the range of 1 × 10^–3^ µM (A549, H358) or 1 × 10^–2^ µM (BEAS-2B) and 30 µM using a WST-1 assay. The impact of cisplatin on the viability of A549 and H358 lung cancer cells was investigated following a 48-h incubation period. This treatment protocol was chosen based on recent findings that demonstrated a 48-h incubation to be sufficient for the induction of migration, viability, and tube formation of HUVECs exposed to conditioned media (CM) from cancer cells [25]. Using these conditions, cisplatin was shown to elicit profound toxic effects on A549 and H358 lung cancer cells that became statistically significant even at concentrations up from 3 µM in A549 cells and 10 µM in H358 cells (Figure 1A, 1B). In line with this data, cell morphology was drastically changed when A549 cells were exposed to 10 or 30 µM cisplatin for 48 h, with many floating cells and adherent cells exhibiting characteristic features of apoptosis such as membrane blebbing (Figure 1A, right). However, a 48-h incubation with cisplatin at concentrations ≤ 1 µM was nontoxic toward A549 and H358 cells (Figure 1A, 1B). The calculated IC_50_ values of cisplatin´s effect on A549 and H358 cell viability were 11.3 µM and 17.8 µM, respectively. Interestingly, the human bronchial epithelial non-cancer cell line, BEAS-2B, was even more sensitive toward the toxic impact of cisplatin, with a calculated IC_50_ value of 2.35 µM (Figure 1C).

### Impact of cisplatin on HUVEC viability and angiogenic features

**Figure 2:**
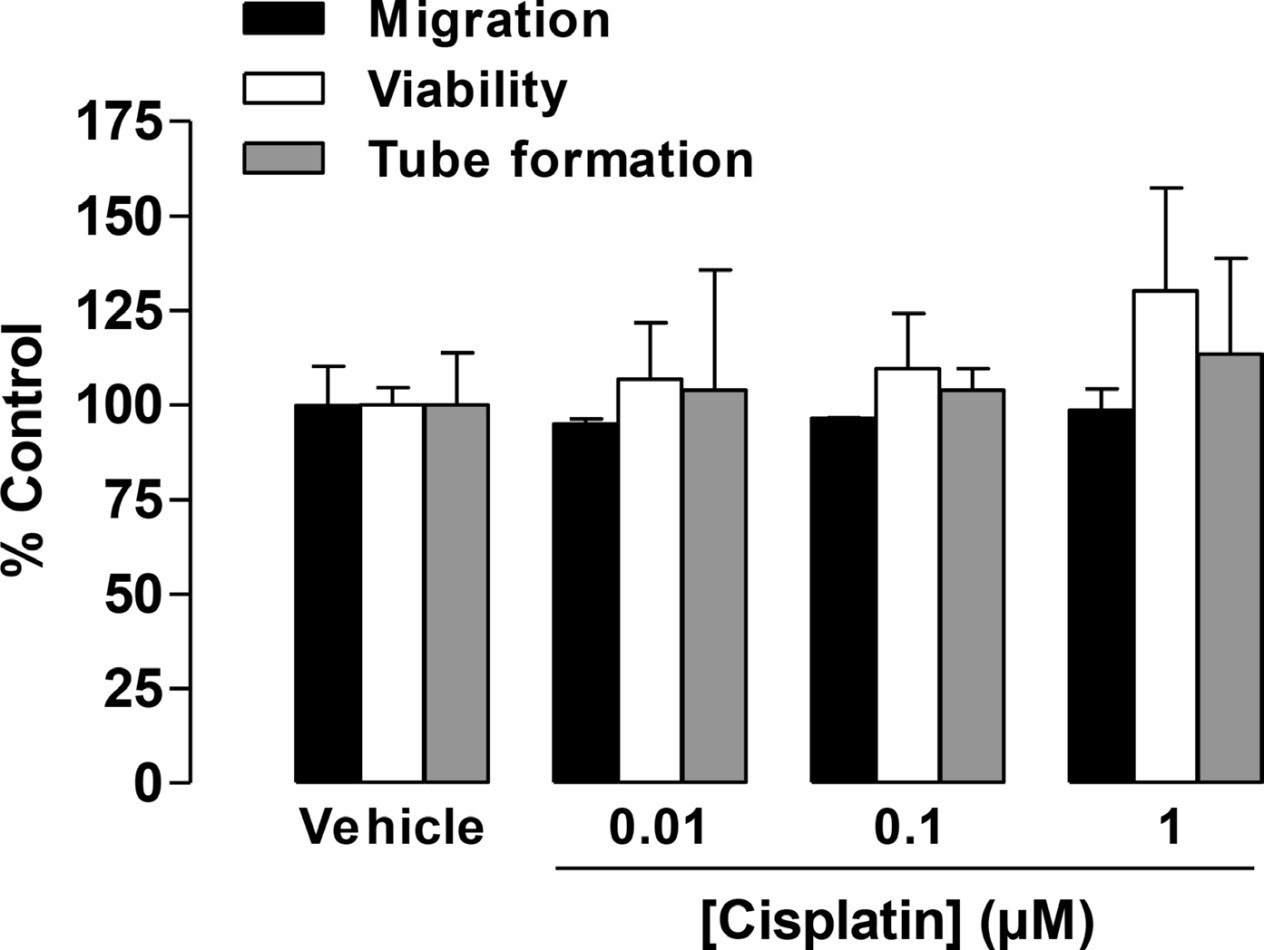
Impact of cisplatin on the migration, viability, and tube formation of HUVECs Migration (black bars, modified Boyden chamber assay) and viability (white bars, WST-1 assay) of HUVECs were measured following a 24-h incubation. Tube formation (grey bars) was determined following a 2-h incubation with vehicle or the indicated concentrations of cisplatin. Percentage of control represents comparison with vehicle-treated cells (set as 100%) in the absence of cisplatin. Values are the mean ± SEM of *n* = 3, respectively. Statistical analyses were performed using one-way ANOVA followed by a *post-hoc* Dunnett test, but statistical significance was not reached between vehicle- and cisplatin-treated cells.

In another set of experiments, the concentration-dependent effect of cisplatin on the viability and migration of HUVECs was tested following a 24-h incubation. Again, this treatment protocol was chosen based on recently published data that demonstrated a 24-h incubation to be sufficient for monitoring the migration and viability of HUVECs in response to CM from lung cancer cells [25]. In HUVECs directly exposed to cisplatin at concentrations between 0.01 µM and 1 µM for 24 h, viability and migration remained virtually unaltered (Figure 2). Similar effects were observed for tube formation when HUVECs were exposed to cisplatin for 2 h (Figure 2).

### CM from cisplatin-treated lung cancer cells confer inhibition of HUVEC migration and tube formation

**Figure 3:**
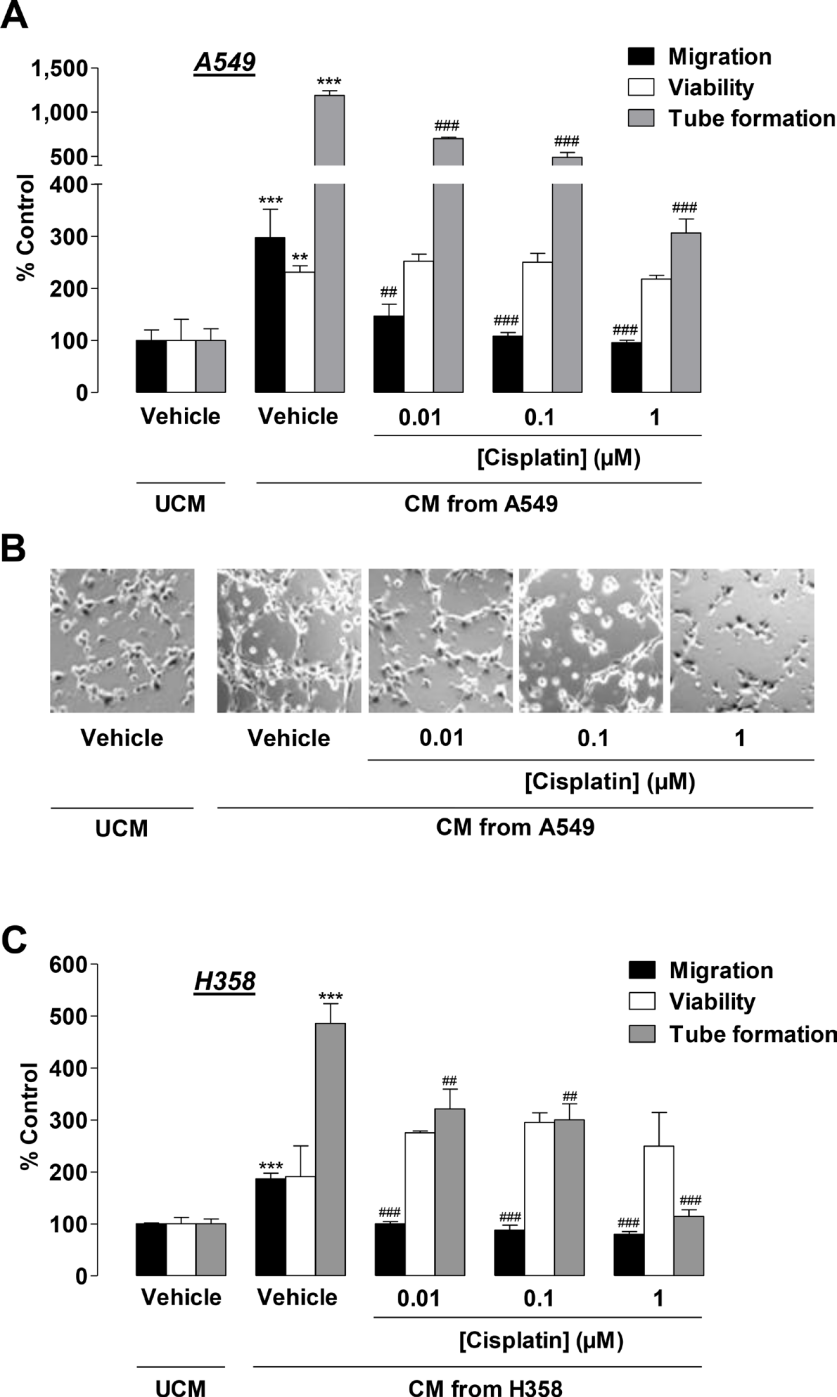
Impact of conditioned media (CM) obtained from cisplatin-treated lung cancer cells on the migration, viability, and tube formation of HUVECs (**A**) and (**C**), Migration (black bars, modified Boyden chamber assay), viability (white bars, WST-1 assay), and tube formation (grey bars) of HUVECs following resuspension in serum-free vehicle-containing DMEM (unconditioned media [UCM]) or CM from vehicle- or cisplatin-treated A549 (**A**) or H358 (**C**) cells. A549 and H358 cells were incubated for 48 h with vehicle or 0.01 µM to 1 µM cisplatin in serum-free DMEM prior to CM being collected for the generation of HUVEC suspensions. Migration and viability of HUVECs were measured following a 24-h incubation of HUVECs suspended in the indicated CM. Tube formation was determined following a 2-h incubation of HUVECs suspended in the indicated CM. (**B**), Phase contrast images of tube formation on the Matrigel layer following a 2-h incubation with the respective treatments. Images are a representative view of HUVECs tested in (**A**). Percentage of control represents comparison with UCM (set as 100%), i.e., vehicle-treated HUVECs in serum-free DMEM in the absence of cisplatin and cancer cells. Values are the mean + SEM of *n* = 6 (**A**, migration) or *n* = 3 (**A**, viability, tube formation, **C**). ^**^p < 0.01, ^***^p < 0.001 vs. vehicle-containing UCM. ^##^ *p* < 0.01, ^###^ *p* < 0.001 vs. CM of vehicle-treated A549 or H358 cells, one-way ANOVA plus a *post-hoc* Bonferroni test.

Subsequently, CM obtained from lung cancer cells were tested for their effects on HUVECs. To this end, A549 cells were incubated with vehicle or cisplatin at concentrations ≤ 1 µM for 48 h. HUVECs suspended in CM from vehicle- or cisplatin-treated A549 cells were seeded on transwell chambers for analysis of migration or on 96-well plates for quantification of viability following an additional 24-h incubation period. For quantification of tube formation, HUVECs suspended in CM from A549 cells were seeded on Matrigel-coated 48-well plates for 2 h. CM from vehicle-treated A549 cells was shown to significantly increase migration, viability, and tube formation of HUVECs as compared with HUVECs exposed to vehicle-containing DMEM, below referred to as unconditioned media (UCM; Figure 3A, second triplet versus first triplet, Figure 3B). HUVECs challenged with CM from A549 cells treated with cisplatin for 48 h exhibited a significant concentration-dependent decrease in migration (Figure 3A, black bars) and tube formation (Figure 3A, grey bars, Figure 3B) as compared with CM from vehicle-treated A549 cells. In contrast, cell viability remained virtually unaltered (Figure 3A, white bars).

To exclude a cell line-specific effect restricted to A549, H358 cells were also treated with vehicle or cisplatin for 48 h prior to CM being collected for HUVEC suspension and subsequent evaluation of angiogenic features. CM from vehicle-treated H358 cells caused a likewise significant increase in HUVEC migration and tube formation as compared with HUVECs exposed to vehicle-containing UCM (Figure 3C, black and grey bars, second triplet versus first triplet). Although an increase in HUVEC viability became obvious, it did not reach statistical significance (Figure 3C, white bars, second triplet versus first triplet). As expected, CM from cisplatin-treated H358 cells similarly conferred inhibition of HUVEC migration (Figure 3C, black bars) and tube formation (Figure 3C, grey bars) as compared with CM from vehicle-treated H358 cells. Again, viability (Figure 3C, white bars) was not significantly altered by all cisplatin concentrations tested.

In another set of experiments, we investigated the migration of HUVECs in a co-culture system denoted as “the non-contact co-culture system” [27]. For this purpose, A549 and H358 cells were treated with vehicle or cisplatin at concentrations ≤ 1 µM for 48 h in 24-well plates. Subsequently, HUVECs were seeded into the cell culture inserts in serum-free DMEM and placed onto the cancer cells to allow migration for a further 24 h. Inhibition of HUVEC migration toward A549 was concentration-dependent, whereas cisplatin-decreased migration of HUVECs toward H358 was a threshold effect (Table 1). Noteworthy, cisplatin in the lower chamber in the absence of lung cancer cells (Table 1, right column) had virtually no effect on HUVEC migration.

**Table 1:** Impact of vehicle- or cisplatin-treated lung cancer cells on the chemoattraction of HUVECs

Cisplatin concentration (µM)	HUVEC migration (% Control)		
	A549	H358	cell-free
0	100.0 ± 3.6	100.0 ± 2.5	100.0 ± 8.4
0.01	64.5 ± 2.6 ^***^	74.9 ± 2.0 ^***^	114.2 ± 1.9
0.1	62.9 ± 1.8 ^***^	71.2 ± 3.4 ^***^	117.9 ± 4.2
1	56.5 ± 1.6 ^***^	75.4 ± 2.4 ^***^	119.4 ± 2.3

Following a 48-h incubation of A549 (left) or H358 (middle) cells with vehicle or 0.01 µM to 1 µM cisplatin in serum-free DMEM, the chemoattraction of HUVECs was measured using modified Boyden chamber assays. Values in the right column refer to experiments with cisplatin (in serum-free DMEM) in the lower chamber in the absence of cancer cells (cell-free). HUVECs were resuspended in serum-free DMEM and loaded onto upper chambers. Subsequently, the upper chambers were placed into the cancer cell-containing companion plates and incubated for a further 24 h prior to the read-out of migration. Values are the mean ± SEM of *n* = 4). ^***^p < 0.001 vs. corresponding vehicle control, one-way ANOVA plus a *post-hoc* Dunnett test.

### Induction of antiangiogenic TIMP-1 by cisplatin

**Figure 4:**
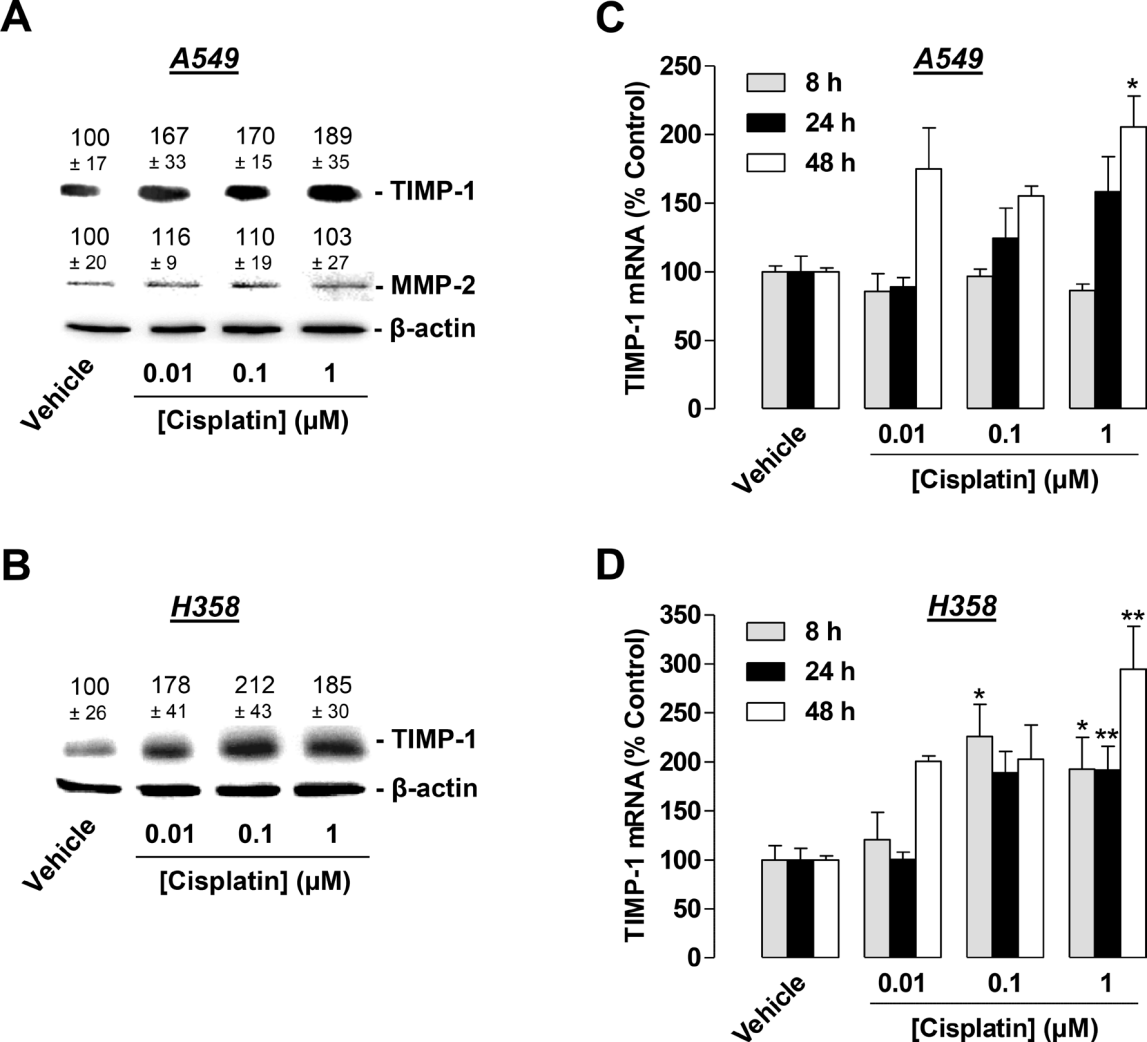
Concentration-dependent effect of cisplatin on TIMP-1 expression in A549 and H358 cells (**A**) and (**B**), Western blotting analysis of TIMP-1 release into conditioned media (CM) of A549 (**A**) or H358 (**B**) cells treated with vehicle or the indicated concentrations of cisplatin for 48 h. Values above the blots are the mean ± SEM and represent alterations in TIMP-1 and MMP-2 protein levels in CM in comparison with vehicle-treated cells (set as 100%), according to densitometric analyses. Western blotting analysis of TIMP-1 and MMP-2 in CM was supplemented with β-actin analyses of the respective cell lysates. (**C**) and (**D**), Real-time RT-PCR analysis of the lysates obtained from A549 (**C**) or H358 (**D**) cells. Cells were incubated with vehicle or cisplatin at the indicated concentrations for 8 h (grey bars), 24 h (black bars), or 48 h (white bars). Percentage of control represents comparison with vehicle-treated cells (set as 100%) in the absence of cisplatin. Values are the mean ± SEM of *n* = 6 (**A**, **B**), *n* = 3–4 (**C**, **D**). ^*^p < 0.05, ^**^p < 0.01 vs. corresponding vehicle control, one-way ANOVA plus a *post-hoc* Dunnett test.

Based on a recent investigation by our group demonstrating that TIMP-1 is released from lung cancer cells as part of the antiangiogenic impact of cannabinoids [25], in addition to previous findings revealing cisplatin to similarly induce TIMP-1 in lung cancer cells [26], we focused on the contribution of TIMP-1 to the antiangiogenic effect of CM from cisplatin-treated A549 and H358 cells on HUVECs. To this end, experiments were performed to address a probable induction of TIMP-1 release from lung cancer cells into the CM in response to a 48-h incubation with 0.01 to 1 µM cisplatin. In accordance with our previous findings demonstrating that 30 µM cisplatin induces TIMP-1 in A549 cells [26], cisplatin caused an increased release of TIMP-1 into CM from A549 at a concentration as low as 0.01 µM (Figure 4A). This finding was confirmed with H358 cells (Figure 4B). Notably, TIMP-1 protein levels in H358 cells exposed to cisplatin exhibited a saturated response at 1 µM rather than a concentration-dependent increase (Figure 4B). In further agreement with our previous data demonstrating MMP-2 expression to be virtually unaltered by 30 µM cisplatin in A549 cells [26], MMP-2 levels remained unchanged in response to cisplatin treatment at 0.01–1 µM in A549 cells (Figure 4A). In H358 cells, however, we were unable to detect MMP-2. Real-time RT-PCR experiments with lysates obtained from A549 or H358 cells exposed to cisplatin for 8, 24, or 48 h revealed an upregulation of TIMP-1 mRNA in both cell lines (Figure 4C, 4D). Noteworthy, the TIMP-1 mRNA induction in both cell lines did not exhibit a consistent concentration-dependent increase, likely due to the pronounced delayed TIMP-1 induction by the lowest concentration (0.01 µM).

### Cisplatin-induced TIMP-1 release from lung cancer cells is causally linked to its antiangiogenic effect on HUVECs

To provide evidence of a causal link between cisplatin-induced TIMP-1 expression and the observed antimigratory and antitube-forming impact of CM from cisplatin-treated lung cancer cells on HUVECs, subsequent experiments were carried out using TIMP-1 siRNA knockdown. HUVECs were exposed to CM obtained from lung cancer cells that were treated with vehicle or 1 µM cisplatin in the presence or absence of TIMP-1 siRNA or a non-silencing siRNA control. Since serum-free vehicle-containing medium (compare UCM groups in Figure 3) does not contain any components released by tumor cells, these reference groups were omitted from experiments that focused on the impact of TIMP-1 release by cancer cells on angiogenic features of HUVECs. The respective values obtained from angiogenic analyses of HUVECs exposed to CM from vehicle-treated lung cancer cells were therefore defined as the 100% control.

**Figure 5:**
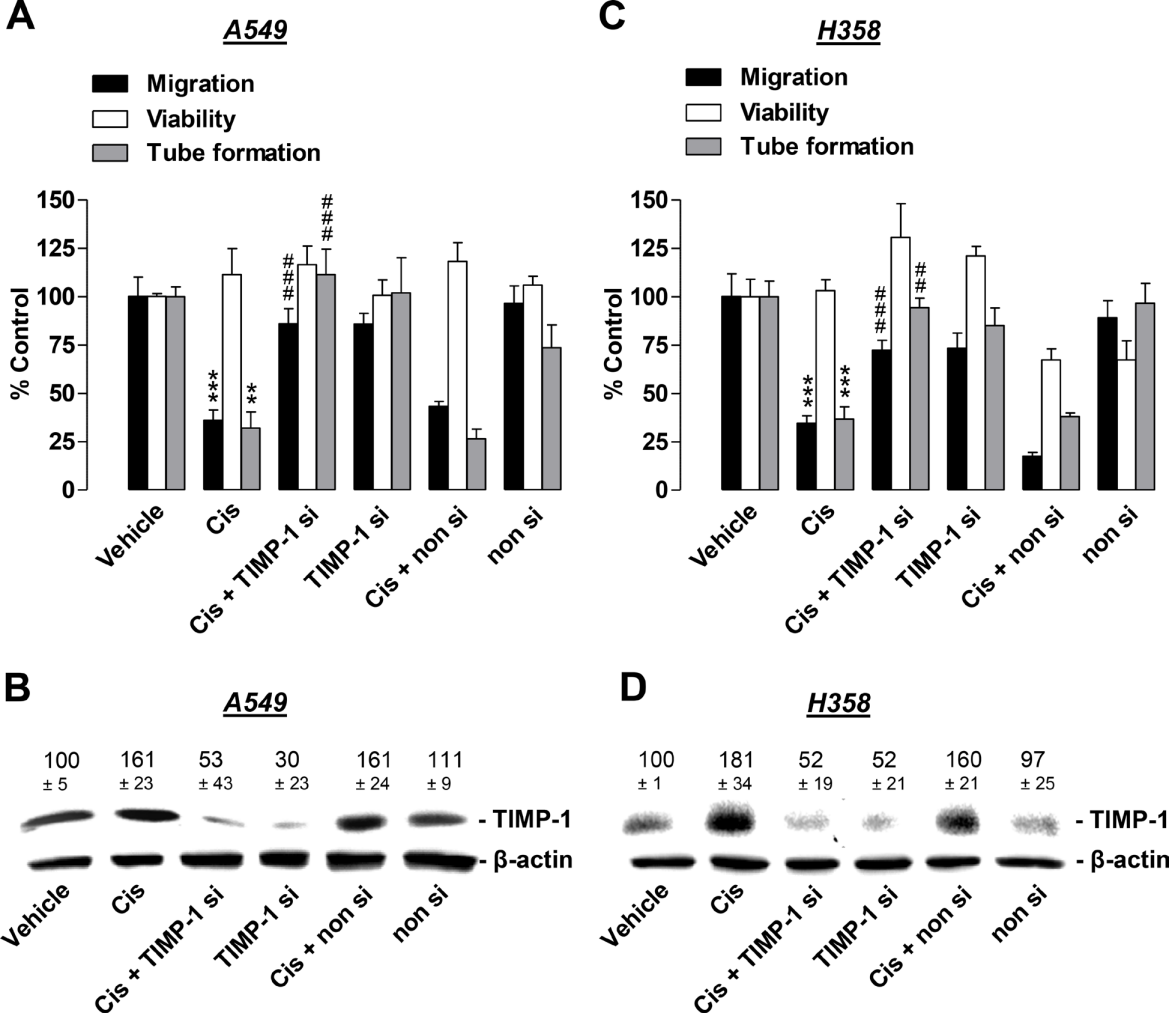
Angiogenic capabilities of HUVECs suspended in conditioned media (CM) from cisplatin- or vehicle-treated A549 and H358 cells in the presence or absence of TIMP-1 siRNA A549 and H358 cells were incubated with transfection reagent in the absence of any siRNA (first and second triplets or bands), transfected with TIMP-1 siRNA (TIMP-1 si; third and fourth triplets or bands), or non-silencing siRNA (non si, fifth and sixth triplet or bands) for 24 h in DMEM containing 10% FCS. Subsequently, cells were washed and treated with vehicle or 1 µM cisplatin for 48 h in serum-free DMEM prior to collection of CM. (**A**) and (**C**), HUVECs suspended in CM from A549 (**A**) or H358 (**C**) cells were subjected to the upper chambers to quantify migration (black bars) or were used in the viability assay (WST-1, white bars) following a further 24-h incubation. Tube formation assays were performed after a 2-h incubation of HUVECs with the indicated CM (grey bars). (**B**) and (**D**), Monitoring of TIMP-1 was performed in parallel using CM obtained from A549 (**B**) or H358 (**D**) cells. Western blot images are representative of each experiment. Values above the blots are the mean ± SEM and represent alterations in TIMP-1 protein levels in CM in comparison with vehicle-treated cells (set as 100%), according to densitometric analyses. Western blotting analysis of TIMP-1 in CM was supplemented with β-actin analysis of the respective cell lysates. Values are the mean ± SEM of *n* = 3 (**A**, migration and tube formation, **B**, **C**), *n* = 4 (**D**) or *n* = 6 (**A**, viability). ^**^p < 0.01, ^***^p < 0.001 vs. corresponding vehicle control; ^##^ *p* < 0.01, ^###^ *p* < 0.001 vs. the respective cisplatin-treated group without siRNA, one-way ANOVA plus a *post-hoc* Bonferroni test.

Our investigations revealed knockdown of cisplatin-induced TIMP-1 in A549 cells to significantly inhibit the antimigratory impact of CM obtained from cisplatin-treated lung cancer cells on HUVECs (Figure 5A, black bars). A functional contribution of TIMP-1 was similarly observed as antitube-forming effects on HUVECs (Figure 5A, grey bars). Conversely, viability was similar to vehicle control levels (Figure 5A, white bars). Using the same treatment protocol, TIMP-1 siRNA was sufficient to profoundly inhibit cisplatin-induced TIMP-1 expression as assessed by western blotting analyses of CM from A549 cells (Figure 5B).

Similar results were obtained from experiments with H358 cells (Figure 5C, 5D). Accordingly, knockdown of TIMP-1 caused a significant suppression of cisplatin-induced inhibition of HUVEC migration (Figure 5C, black bars) and tube formation (Figure 5C, grey bars), whereas viability was not significantly altered (Figure 5C, white bars). Western blotting experiments monitoring the knockdown efficiency using equivalent conditions to those used for A549 cells revealed H358 cells to also be sensitive toward siRNA transfection, resulting in a substantial knockdown of cisplatin-induced TIMP-1 expression (Figure 5D).

### Mitogen-activated protein kinases confer cisplatin-induced TIMP-1 release from lung cancer cells and the subsequent antiangiogenic impact on HUVECs

**Figure 6:**
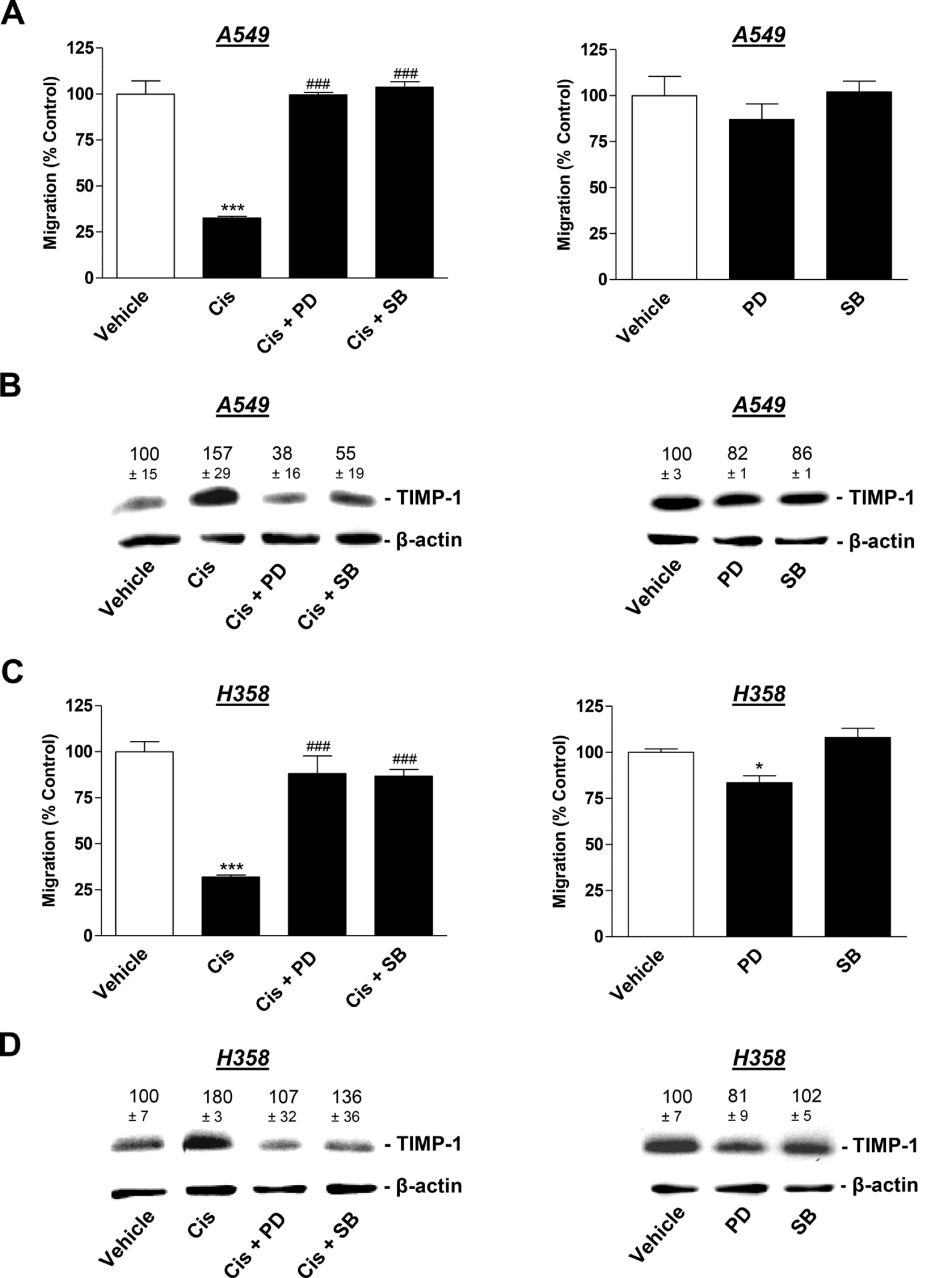
Influence of MAPK inhibitors on the angiogenic capabilities of HUVECs suspended in conditioned media (CM) from cisplatin- or vehicle-treated A549 and H358 cells A549 and H358 cells were preincubated with vehicle, the p38 MAPK inhibitor, SB203580, or the inhibitor of p42/44 MAPK activation, PD98059, (10 µM each) for 1 h. Subsequently, cells were treated with vehicle or 1 µM cisplatin for 48 h prior to the collection of CM for the analysis of TIMP-1 (**B** and **D**) or for the preparation of HUVEC suspension that was subsequently subjected to the upper chamber for quantification of migration following a further 24-h incubation (**A** and **C**). Western blotting images are representative of each experiment. Values above the blots are the mean ± SEM and represent alterations in TIMP-1 protein levels in CM in comparison with vehicle-treated cells (set as 100%), according to densitometric analyses. Western blotting analysis of TIMP-1 in CM was supplemented with β-actin analysis of the respective cell lysates. Values are the mean ± SEM of *n* = 4 except of *n* = 3 in (**D**), left. ^*^p < 0.05, ^***^p < 0.001 vs. corresponding vehicle control; ^###^ *p* < 0.001 vs. the respective cisplatin-treated group, one-way ANOVA plus a *post-hoc* Bonferroni test.

Based on the results of a recent investigation reporting cisplatin-induced TIMP-1 expression to be dependent on a pathway involving the activation of p38 and p42/44 mitogen-activated protein kinases (MAPK) [26], these targets were further focused on using the respective kinase inhibitors. To this end, A549 and H358 cells were pretreated with the p38 MAPK inhibitor, SB203580, or the inhibitor of p42/44 MAPK activation, PD98059, in the presence or absence of cisplatin. Both SB203580 and PD98059 reversed the inhibitory effect of CM from cisplatin-treated lung cancer cells on HUVEC migration (Figure 6A and 6C, left). In accordance with the proposed role of TIMP-1 in this context, inhibition of the p38 and p42/44 MAPK pathways also conferred inhibition of cisplatin-induced TIMP-1 release by A549 (Figure 6B, left) and H358 cells (Figure 6D, left). CM from A549 exposed to SB203580 and PD98059 in the absence of cisplatin did not virtually alter the migratory potential of HUVEC (Figure 6A, right) or TIMP-1 expression in A549 cells (Figure 6B, right). Similar results were obtained from experiments using H358 cells (Figure 6C, 6D, right). Although the effect of PD98059 on migration yielded statistical significance, the respective decrease was comparably low.

### CM from cisplatin-treated BEAS-2B bronchial epithelial cells does not alter HUVEC migration, tube formation, or viability

To evaluate whether the observed antiangiogenic effects were restricted to a cancer cell microenvironment, the same experimental setting was used to perform experiments with non-cancer cells. For this purpose, BEAS-2B, a cell line established from the bronchial epithelium of individuals without cancer [28], which was therefore assigned as “a normal lung epithelial cell line” [29, 30], was used for the preparation of CM.

**Figure 7:**
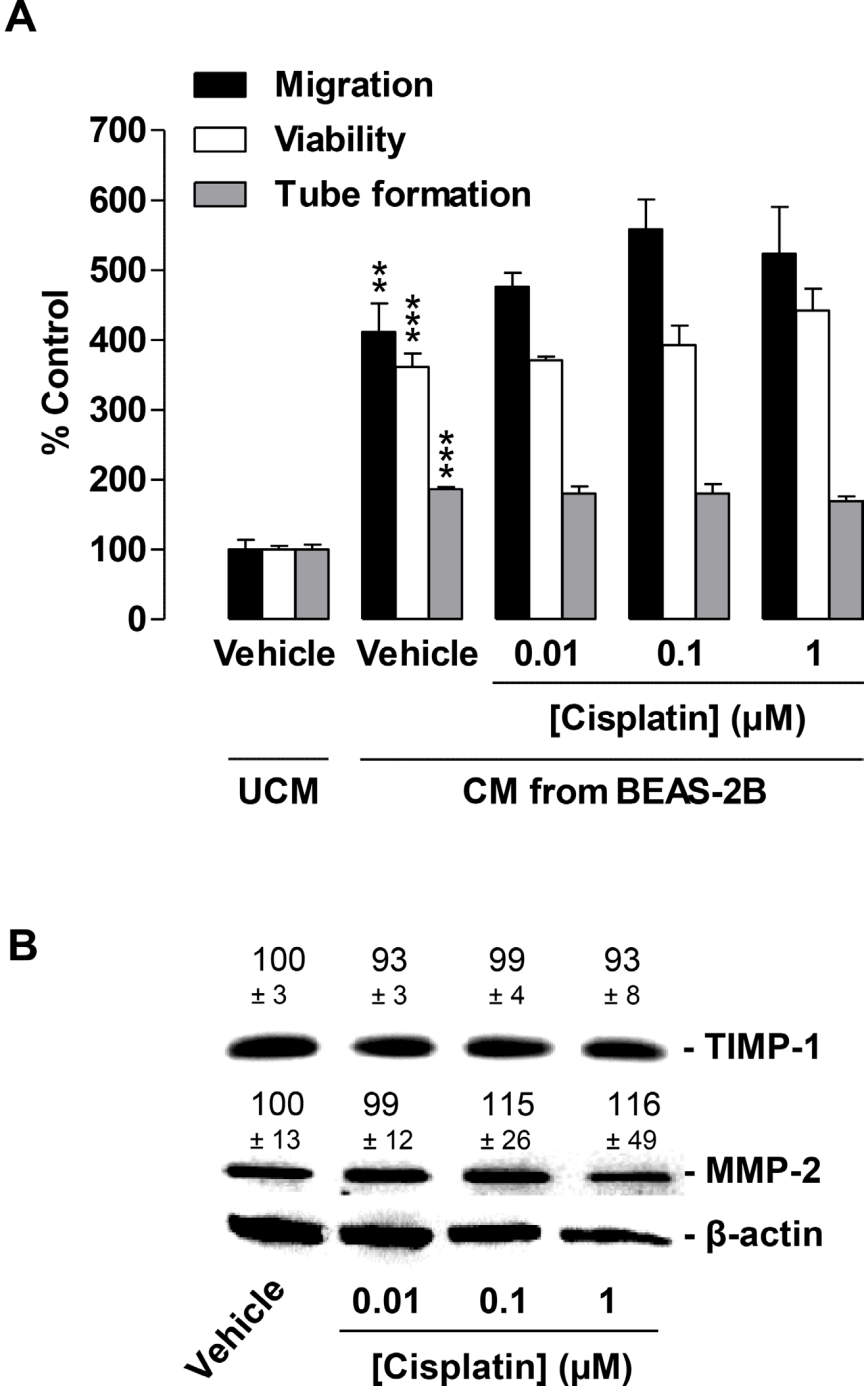
Impact of conditioned media (CM) obtained from cisplatin-treated non-cancer bronchial epithelial BEAS-2B cells on migration, viability, and tube formation of HUVECs (**A**), Migration (black bars, modified Boyden chamber assay), viability (white bars, WST-1 assay) and tube formation (grey bars) of HUVECs following resuspension in serum-free vehicle-containing DMEM (unconditioned media [UCM]) or CM from vehicle- or cisplatin-treated BEAS-2B cells. BEAS-2B cells were incubated for 48 h with vehicle or the indicated concentrations of cisplatin. Incubation time of HUVECs was 24 h in migration and viability assays. Tube formation was measured following a 2-h exposure of HUVECs to CM. Percentage of control represents comparison with UCM (set as 100%), i.e., vehicle-treated HUVECs in serum-free DMEM in the absence of cisplatin and BEAS-2B cells. (**B**), Western blotting analysis of TIMP-1 and MMP-2 release into CM of BEAS-2B cells treated with vehicle or the indicated concentrations of cisplatin for 48 h. Western blot images are representative of each experiment. Values above the blots are the mean ± SEM and represent alterations in TIMP-1 and MMP-2 protein levels in CM in comparison with vehicle-treated cells (set as 100%), according to densitometric analyses. Western blotting analysis of CM was supplemented with β-actin analysis of the respective cell lysates. Values are the mean ± SEM of *n* = 3 (**A**) or *n* = 6 (**B**). ^**^p < 0.01; ^***^p < 0.001 vs. corresponding vehicle control, one-way ANOVA plus a *post-hoc* Bonferroni test. Statistical analyses did not yield significant differences between cisplatin-treated groups (third, fourth, or fifth triplet) and the corresponding vehicle-treated group (second triplet). For reasons of clarity, statistical differences (all at *p* < 0.001) between first versus third, fourth, or fifth triplet are not indicated.

Similar to CM from cancer cells, CM from vehicle-treated BEAS-2B cells was found to significantly increase HUVEC migration, viability, and tube formation as compared with vehicle-containing UCM (Figure 7, second triplet versus first triplet). However, in contrast to the reduction in migration and tube formation by CM obtained from cisplatin-treated cancer cells, CM from BEAS-2B challenged with the same concentrations of cisplatin for 48 h had virtually no effect on HUVEC migration (Figure 7A, black bars) or tube formation (Figure 7A, grey bars). In addition, viability (Figure 7A, white bars) was also unchanged.

In agreement with the proposed inhibitory effect of TIMP-1 on HUVEC migration and tube formation, cisplatin did not alter TIMP-1 levels in CM obtained from BEAS-2B cells following a 48-h incubation period (Figure 7B). In the same experimental setting, MMP-2 also remained virtually unaltered when BEAS-2B cells were exposed to cisplatin (Figure 7B).

## DISCUSSION

Recent studies have reported the antiangiogenic effects of cisplatin as an important factor contributing to its tumor-regressive action. However, publications that address the mechanism of low-dose cisplatin on the angiogenic capability of endothelial cells are rare. To date, results from these evaluations have mainly focused on the regulation of parameters linked to angiogenic processes sparing considerations of a potential tumor-to-endothelial communication. The data obtained from the present investigation provide first-time proof for cisplatin-induced TIMP-1 release from NSCLC cells as a specific cancer-associated antiangiogenic action on endothelial cells.

There are several lines of evidence supporting this notion. Firstly, CM obtained from cisplatin-treated A549 and H358 lung cancer cells were shown to cause a significant inhibition of HUVEC migration and tube formation. Secondly, knockdown of cisplatin-induced TIMP-1 expression was found to significantly attenuate the inhibition of HUVEC migration and tube formation by CM from both NSCLC cell lines challenged with cisplatin. Thirdly, blockade of the cisplatin-induced TIMP-1 release by inhibitors of MAPK signalling pathways likewise conferred a reversal of the inhibitory impact of CM from cisplatin-treated lung cancer cells. Fourthly, the observed antiangiogenic impact of cisplatin cannot be ascribed to a toxic effect since the tested cisplatin concentrations ranging from 0.01 µM and 1 µM did not cause decreased viability of either the cancer or endothelial cells. Fifthly, the observed antiangiogenic effects were not mediated via a direct action of cisplatin toward endothelial cells. Accordingly, direct exposure of HUVECs to cisplatin at concentrations up to 1 µM had virtually no effect on HUVEC migration, viability, or tube formation. Finally, the TIMP-1-inducing, and therefore antiangiogenic, impact of cisplatin could not be reproduced using CM from non-cancer bronchial epithelial cells. The current data, therefore, suggest that the indirect antiangiogenic action of cisplatin at concentrations ≤ 1 µM strictly depends on the presence of cancer cells.

The data presented here are in line with other reports that have also found cisplatin at 1 µM to leave migration and tube formation of HUVEC virtually unaltered, when HUVEC were directly exposed to the substance [7, 31]. However, it cannot be excluded that higher concentrations of cisplatin, which were not tested in the present investigation, may directly inhibit endothelial activation in our experimental system. With respect to direct antiangiogenic effects of higher cisplatin concentrations toward HUVECs, contradictory results have been reported. On one hand, cisplatin was found to have virtually no effect on HUVEC migration or tube formation when tested at a concentration of 10 µM [24]. On the other hand, a recent investigation found that cisplatin at 10 µM decreased the viability of HUVECs with an apparent IC_50_ value of 2.35 µM, inhibited migration, and decreased the activities of matrix metalloproteinases (MMP)-1 and -2 [7]. Inhibition of HUVEC migration associated with the downregulation of MMP-2 was also reported in another study testing cisplatin at concentrations up to 80 µM [32]. However, the clinical relevance of *in vitro* data obtained with cisplatin at concentrations up to 100 µM was recently criticized [33] in view of the fact that intravenous bolus injections of cisplatin administered in humans at maximum tolerated doses of 100 mg/m^2^ elicited total plasma levels of unbound cisplatin of 10.9 µM with a half-life of < 1 h [20]. Collectively, discrepancies in the findings may at least in part be explained by differences in experimental details. Considering that cisplatin at 1 µM has been demonstrated to elicit moderate toxic effects on VEGF-stimulated human dermal microvascular cells (HDMEC) [34], it is tempting to speculate that the results of such analyses are dependent on the endothelial cell type.

In previous years, TIMP-1 has been demonstrated to be a pivotal antiangiogenic factor in numerous biological systems [35–40]. Recently, this notion was substantiated by experiments from our group revealing that recombinant TIMP-1 inhibits migration and tube formation of HUVECs [25]. However, the exact mechanism by which TIMP-1 released from cancer cells causes inhibition of migration and tube formation of HUVECs remains to be clarified. On one hand, the inhibition of MMP-2, a major target of the inhibitory action of TIMP-1, may confer blockade of HUVEC migration by cisplatin as previously described [32]. On the other hand, several investigations have reported TIMP-1 to elicit effects independent of its proteolysis altering actions [41–44]. Another possible mechanism of action of TIMP-1 may be the inhibition of MMP-9, knockdown of which has previously been found to provide a cellular switch from a migratory to a stationary phase via paxillin/RhoA-dependent cytoskeletal reorganization and stabilization of the β-catenin/E-cadherin complex [45]. In accordance with a proteolysis-independent action, several groups have found alterations in migration elicited via modulation of the MMP/TIMP system even when uncoated chambers were used [45–47].

Interestingly, a recent report found HUVEC migration to be virtually unaltered when CM from A549 cells treated with 5 µM cisplatin was used as the chemoattractant in the lower companion plate [48]. This is in accordance with the results from our present experiments where cisplatin added to the lower chamber of a modified Boyden chamber system as the chemoattractant, without cancer cells, did not exert an inhibitory effect on HUVEC migration. With respect to these data, the findings of the present study using CM from cisplatin-treated lung cancer cells in the upper chamber favor the hypothesis that cisplatin acting via increased TIMP-1 release most likely confers interference with cellular motility rather than with chemoattraction of endothelial cells. Notably, when using vehicle- or cisplatin-treated A549 and H358 cells in the lower chamber as the chemoattractant, we also observed an inhibitory effect of cisplatin on the migration of HUVECs suspended in serum-free DMEM. This effect may be due to the impaired motility as a result of TIMP-1 entering the upper chamber during the 24-h migration phase of the experiment. However, the inhibitory effect of cisplatin in this experimental setting did not yield the efficacy as compared with the conventional setting using HUVECs suspended in CM on the upper chamber. One possible explanation is that TIMP-1 released by cancer cells during the prior 48-h cisplatin treatment more effectively blocks migration if HUVECs are immediately exposed to the total TIMP-1 content of CM prior to being subjected to the upper chamber.

Noteworthy, in the present study, the basal release of TIMP-1 from vehicle-treated A549 or H358 cells had no influence on basal CM-induced angiogenic features of HUVECs. Accordingly, TIMP-1 siRNA knockdown, which caused partial inhibition of basal TIMP-1 expression, did not further increase migration, viability, or tube formation of HUVECs as could have been expected from the antiangiogenic effect of TIMP-1.

Since angiogenesis is a multistep mechanism, additional intercellular regulations involved in cisplatin-induced antiangiogenic responses appear feasible. Accordingly, MMP-2 downregulation does not exclusively occur in endothelial cells [6, 30], but has also been shown to occur in lysates and culture media from cancer cells such as glioblastoma cells exposed to 25 µM cisplatin [49]. However, the effects of cisplatin on members of the MMP family in cancer cells are obviously inconsistent. In a study by our group revealing TIMP-1 induction as a crucial factor in the anti-invasive action of cisplatin on cervical carcinoma cell lines, as well as on A549 cells, cisplatin at 30 µM had virtually no effect on MMP-2 expression [26], which is in line with the experiments regarding MMP-2 regulation by cisplatin in A549 cells presented here. Noteworthy, in this previous study, 30 µM cisplatin caused no significant alterations in MMP-9 or TIMP-2 expression in cervical carcinoma cells or in TIMP-2 expression in A549 cells. On the other hand, *in vivo* experiments using a rodent model of liver cancer demonstrated that LDM treatment with cisplatin resulted in reduced levels of VEGF and MMP-2 [21]. Downregulation of VEGF as a possible mechanism underlying the antiangiogenic action of cisplatin was furthermore validated in ovarian cancer xenografts [8]. Furthermore, decreases in VEGF and hypoxia-inducible factor 1α (HIF-1α), a major transcription factor implicated in tumor neovascularization, were found to be associated with a cisplatin-induced reduction in vessel density in a Lewis lung carcinoma model [50]. A contribution of the HIF-1α/VEGF axis to cisplatin-induced antiangiogenesis was additionally reported for lung cancer cell lines containing exon 19 deletions in the epidermal growth factor receptor (EGFR) [13]. However, with respect to the data presented here, a probable contribution of cisplatin-induced VEGF downregulation to antiangiogenesis appears unlikely since a previous study could not detect VEGF downregulation in A549 xenografts of cisplatin-treated mice [14]. In the latter investigation, cisplatin reduced tumor neovascularization despite the lack of VEGF-lowering properties, suggesting alternative mechanisms bypassing alterations in VEGF. In agreement with this hypothesis, another study observed no alterations in VEGF serum levels in lung cancer patients receiving LDM chemotherapy with cisplatin [51].

Finally, there also exist certain studies that observed no antiangiogenic effects of cisplatin *in vivo*. As such, cisplatin did not elicit an antiangiogenic response in mice xenografted with H358 [52], which contradicts our findings with the same cell line. However, in the cited study, mice were treated with 5 mg/kg cisplatin only once per week without yielding growth inhibitory effects on H358 xenografts within a period of 22 days. Thus, both the dose and schedule may be insufficient to yield inhibition of angiogenesis *in vivo*, probably due to the short half-life of cisplatin [20]. Similarly, cisplatin treatment at 0.25 mg/kg twice per week did not confer inhibition of tumor angiogenesis in a rat bladder cancer model [53]. Another study found that 5 mg/kg cisplatin administered 3 times per week had no effect on angiogenesis in mice transplanted with reassembled tumors consisting of HDMECs and an oral squamous cell carcinoma cell line embedded in Matrigel [34]. Conversely, a weekly treatment of mice with 4 mg/kg cisplatin over 3 weeks was sufficient to inhibit tumor angiogenesis in a xenograft model of ovarian cancer [11]. Thus, the antiangiogenic efficacy of cisplatin apparently depends on dosage, frequency of administration, cancer type, and specifics of the experimental setting.

With respect to the broad spectrum of severe side effects of cisplatin when used at high dosage, LDM regimens have recently gained attention. In fact, LDM chemotherapy targeted at inhibiting tumor neovascularization with reduced toxicity in healthy tissue may be an effective and safe treatment option for drug-resistant cancers [54, 55]. The mechanisms involved in such treatment regimes are currently under intensive investigation. In addition to demonstrating antiangiogenic effects of low cisplatin concentrations, the present data also argue in favor of low-dose treatment regimes in view of the remarkable toxicity of cisplatin toward the non-cancer bronchial epithelial cell line, BEAS-2B.

Collectively, the data presented here suggest that cisplatin-induced TIMP-1 release from cancer but not non-cancer cells is a crucial event that causes a reduction in the angiogenic capability of endothelial cells. This cisplatin-triggered cell-to-cell communication, resulting in the inhibition of angiogenic features of endothelial cells, may represent a novel mechanism by which lower, non-toxic concentrations of cisplatin may exert anticarcinogenic effects at a clinically relevant level.

## MATERIALS AND METHODS

### Materials

Aprotinin, cisplatin (cis-diamminedichloroplatinum(II)), p-coumaric acid, ethanol, N, N-dimethylformamide (DMF), luminol, orthovanadate, and phenylmethylsulfonyl fluoride (PMSF) were obtained from Sigma-Aldrich (Taufkirchen, Germany). Dulbecco’s modified Eagle’s medium (DMEM) with 4 mM L-glutamine and 4.5 mg/ml glucose was obtained from Lonza (Cologne, Germany). Fetal calf serum (FCS) and phosphate-buffered saline (PBS) were purchased from PAN Biotech (Aidenbach, Germany). Leupeptin was purchased from Biomol (Hamburg, Germany). Dimethyl sulfoxide (DMSO), ethylenediaminetetraacetic acid (EDTA), glycerol, hydrogen peroxide (H_2_O_2_), sodium chloride (NaCl), Tris hydrochloride (Tris-HCl), and Tris ultrapure were purchased from AppliChem (Darmstadt, Germany). PD98059 was purchased from Tocris Bioscience (Wiesbaden-Nordenstadt, Germany), and penicillin-streptomycin was bought from Invitrogen (Darmstadt, Germany). 4-(2-hydroxyethyl)-1-piperazineethanesulfonic acid (HEPES) was obtained from Ferak (Berlin, Germany), and SB203580 was purchased from Enzo Life Sciences (Lörrach, Germany). Triton^®^ X-100 was bought from Roth (Karlsruhe, Germany).

### Cell culture

A549 lung carcinoma cells were obtained from the Deutsche Sammlung von Mikroorganismen und Zellkulturen GmbH (Braunschweig, Germany; DSMZ no.: ACC 107). NCl-H358 cells (assigned as H358) and the human bronchial epithelial cell line, BEAS-2B, were obtained from the American Type Culture Collection (ATCC^®^; Wesel, Germany, ATCC no.: CRL-5807™ [H358], ATCC no.: CRL-9609™ [BEAS-2B]). A549, H358, and BEAS-2B cells were maintained in DMEM supplemented with 10% (v/v) heat-inactivated FCS, 100 U/ml penicillin and 100 µg/ml streptomycin. HUVECs were obtained from PromoCell (Heidelberg, Germany) and cultivated using the Endothelial Cell Growth Medium Kit (C-22110) from the same company. For experiments, HUVECs were used between passages 2 and 7.

### Treatment protocol for the evaluation of indirect cisplatin effects on HUVEC migration, viability, and tube formation

For the evaluation of the effect of CM obtained from A549, H358, or BEAS-2B cells on HUVEC migration, viability, and tube formation, cells were seeded at a density of 1 × 10^5^, grown to confluence, and subsequently treated with vehicle or cisplatin in a final volume of 300 µl serum-free DMEM for 48 h on 48-well plates. Test substances were dissolved in DMF (cisplatin) or DMSO (SB203580, PD98059) and diluted with PBS to yield a final concentration of 0.1% (v/v) DMF or 0.1% (v/v) DMSO in the respective experimental settings. As a vehicle control, PBS containing the respective concentrations of DMF or DMSO was used. Following incubation of the cells with vehicle or test substances in serum-free media, CM were collected, centrifuged at 1300 × g for 5 min, and intermediately stored on ice. Meanwhile, HUVECs were washed, trypsinized, and counted. Following removal of the supernatants, HUVECs were resuspended in the respective CM. 300 µl of the resulting HUVEC suspensions containing the pre-adjusted number of 1 × 10^5^ HUVECs were seeded onto the upper chamber of a modified Boyden chamber system for evaluation of migration. For viability assays, 5 × 10^3^ HUVECs were seeded in 100 µl CM on 96-well plates and incubated for 24 h prior to measurement of viability using the WST-1 assay. 5 × 10^4^ HUVECs suspended in 200 µl CM were seeded onto the Matrigel layers on 48-well plates for the tube formation assay. Since serum-free medium devoid of prior contact with cells (indicated as UCM in Figures 3 and 7A) does not contain any components released by tumor cells for western blotting analyses of TIMP-1 (Figures 5B, 5D, 6B, 6D), this reference group was omitted from the respective experiments monitoring migration, viability, and tube formation (Figures 5A, 5C, 6A and 6C).

### Analysis of cellular viability

Cellular viability was determined using the colorimetric WST-1 assay (Sigma-Aldrich). This cell viability test is based on the cleavage of the tetrazolium salt, WST-1 (4-[3-(4-Iodophenyl)-2-(4-nitrophenyl)-2*H*-5-tetrazolio]-1.3-benzene disulfonate), by mitochondrial succinate-tetrazolium-reductase in metabolically active cells. For assessment of HUVEC viability following direct exposure to cisplatin, HUVECs seeded at a density of 5 × 10^3^ cells per well on 96-well plates were allowed to adhere for 6 h in Endothelial Cell Growth Medium (PromoCell) (Figure 2). Subsequently, cells were washed and incubated with vehicle or test substances in serum-free DMEM for 24 h. For viability tests using A549, H358, or BEAS-2B cells (Figure 1), 1 × 10^4^ cells were seeded onto 96-well plates in DMEM containing 10% FCS. After 24 h, media were removed, cells were washed with PBS, and subsequently stimulated with the indicated concentrations of cisplatin for 48 h in serum-free DMEM. To evaluate the effects of CM from A549, H358, or BEAS-2B cells on the viability of HUVECs (Figures 3A, 3C, 5A, 5C, 7A), cells were treated according to the aforementioned treatment protocols.

### Migration assay

The effect of test substances on the migration of HUVECs was determined using uncoated Falcon^®^ Cell Culture Inserts (Corning Inc., Corning, NY, USA) in 24-well plates as previously described [25]. In this modified Boyden chamber assay, cellular motility is monitored by transmigration through pores with a diameter of 8 µm towards a chemoattractant. Briefly, after resuspension of 1 × 10^5^ HUVECs per sample in CM obtained from lung cancer or BEAS-2B cells, the HUVEC/CM suspensions were seeded onto the upper chambers. DMEM containing 10% FCS was added as a chemoattractant to the lower companion plate. Neither conditioned media nor unconditioned media (UCM) contained serum.

For assessment of the direct effects of cisplatin on HUVEC migration (Figure 2), cells were suspended in serum-free DMEM containing vehicle or the indicated concentrations of cisplatin. Thereafter, DMEM containing 10% FCS, serving as a chemoattractant, was loaded into the lower companion plate, and the incubations continued for a further 24 h. Finally, the stationary cells on the upper surface of the inserts were removed with a cotton swab. For calculation of migration, the viability of the migrated cells adhering to the lower sides of uncoated cell culture inserts was determined using the "WST-1 assay".

### Non-contact co-cultures

Additionally, we tested the migration of HUVECs in a non-contact co-culture system using cell culture inserts. For this purpose, A549 and H358 cells were seeded at a density of 1 × 10^5^ cells and grown to confluence in DMEM containing 10% (v/v) FCS for 24 h on 24-well plates. Subsequently, A549 and H358 cells were washed with PBS and treated with vehicle or cisplatin in a final volume of 300 µl serum-free DMEM for 48 h. Thereafter, HUVECs seeded into the upper chambers in serum-free DMEM at a density of 1 × 10^5^ HUVECs per insert were placed into a 24-well plate containing the pretreated cancer cells to allow for migration over a further 24 h.

### Tube formation assay

The tube formation assay is based on the finding that *in vitro* organisation of endothelial cells into capillary-like networks on Matrigel layers mimics cellular behavior of an angiogenic process *in vivo* [56]. To visualize (Figure 3B) and quantify the angiogenic potential of cisplatin or CM obtained from A549, H358, or BEAS-2B cells on HUVECs, tube formation assays were performed on Matrigel-coated 48-well plates as described previously, with slight modifications [25]. In brief, 48-well plates were coated with 30 µl per well ice-chilled Matrigel^®^ Matrix Basement Membrane (BD Biosciences) and allowed to polymerize at 37° C for 1 h.

HUVECs were resuspended in serum-free DMEM containing vehicle (indicated as UCM in Figures 3 and 7A), in serum-free CM from A549, H358, or BEAS-2B cells (Figures 3, 5A, 5C, 6A, 6C, and 7A) or in serum-free DMEM containing vehicle or cisplatin (Figure 2) and seeded at a density of 5 × 10^4^ cells in a volume of 200 µl per well onto Matrigel-coated 48-well plates. For similar experiments previously published by our group, an incubation time of 24 h was used for tube formation assays [25]. As in the present study a 2-h incubation was sufficient for HUVEC to form closed intersections, this incubation time was chosen for read-out. Tube formation was photographed and quantitatively analyzed in total microscopic fields by counting the number of tube-like structures forming closed intersections in an investigator-blinded fashion.

### Western blotting analysis

For analysis of protein levels, A549, H358, and BEAS-2B cells were seeded onto 48-well plates at a density of 1 × 10^5^ cells per well (Figures 4A, 4B, 5B, 5D, 6B, 6D and 7B). Subsequently, cells were washed and incubated with vehicle or cisplatin in serum-free DMEM (Figures 4A, 4B, 6B, 6D and 7B) or transfected according to the protocol mentioned under “siRNA transfection” (Figure 5B, 5D). Following a 48-h incubation period, CM were used for western blotting analyses of TIMP-1 and MMP-2, and the respective cell lysates were used for further analysis of β-actin.

CM were collected, centrifuged at 1300 × g for 5 min, and used for subsequent western blotting analysis of TIMP-1 and MMP-2. For analysis of β-actin, cells were lysed in solubilization buffer (50 mM HEPES pH 7.4, 150 mM NaCl, 1 mM EDTA, 1% [v/v] Triton^®^ X-100, 10% [v/v] glycerol, 1 mM PMSF, 1 mM orthovanadate, 1 µg/ml leupeptin, 10 µg/ml aprotinin) and centrifuged at 10,000 × g for 5 min. Supernatants were used for western blotting analysis.

Total protein amounts of cell lysates were determined using the bicinchoninic acid assay (Pierce, Rockford, IL, USA). Equal amounts of protein from cell lysates (β-actin) and equal volumes of CM (TIMP-1, MMP-2) were separated using 10% sodium dodecyl sulfate-polyacrylamide (Applichem) gels and subsequently transferred to nitrocellulose membranes (Roth), which were then blocked in 5% Blotting Grade Blocker (BioRad, Munich, Germany). Due to the lack of a housekeeping protein secreted into CM, TIMP-1 western blotting analysis of CM was supplemented with β-actin analysis of the respective cell lysates to monitor possible toxic effects.

Blots were probed with specific primary antibodies raised against β-actin (Sigma-Aldrich), TIMP-1 or MMP-2 (Merck KGaA, Darmstadt, Germany). Subsequently, membranes were washed and probed with a horseradish peroxidase-conjugated Fab-specific anti-mouse IgG from Cell Signaling Technology Europe (Leiden, Netherlands). Antibody binding was visualized using a chemiluminescence solution (100 mM Tris-HCl pH 8.5, 1.25 mM luminol, 200 µM p-coumaric acid, 0.09% [v/v] H_2_O_2_, 0.0072% [v/v] DMSO).

### siRNA transfection

siRNA transfection was performed using the Lipofectamine™ RNAiMAX reagent (Thermo Fisher Scientific, Schwerte, Germany) according to the manufacturer’s manual indicated under “Reverse Transfection” with slight modifications. RNAi-Lipofectamine™ RNAiMAX complexes were prepared in Opti-MEM^®^ I Reduced-Serum Medium containing 0.2 µl Lipofectamine™ RNAiMAX per 100 µl. Subsequently, RNA suspension buffer without siRNA (first and second triplet in Figure 5A and 5C; first and second lane in the western blots in Figure 5B and 5D), with TIMP-1 siRNA (Qiagen, Hilden, Germany), or with a non-silencing control sequence siRNA (nonsi; Eurogentec [Cologne, Germany]) were added. Dilutions were mixed, 100 µl was preloaded into each well of a 48-well plate, and incubated for 10–20 min. Finally, 1 × 10^5^ A549 or H358 cells suspended in a volume of 500 µl DMEM containing 10% FCS were added to each well, yielding a final concentration of 2 nM TIMP-1 siRNA and non-silencing siRNA, respectively. Following a 24-h incubation period, cells were washed and treated with vehicle or cisplatin in 300 µl serum free-DMEM. After a further 48 h, CM were collected, centrifuged at 1300 × g for 5 min, and used to either prepare HUVEC suspensions for subsequent functional assays or to perform western blotting analysis of TIMP-1. For analysis of β-actin, cell lysates were used according to the protocol indicated under “Western blotting analysis”.

### Quantitative real-time RT-PCR

Quantitative analysis of mRNA was carried out as described previously [57], with slight modifications. Briefly, 2.5 × 10^4^ cells were seeded onto 24-well plates in DMEM containing 10% FCS. After 24 h, cells were washed and stimulated with vehicle or the indicated concentrations of cisplatin for 8 h, 24 h, or 48 h. Total RNA was isolated using the RNeasy Mini Kit (Qiagen GmbH). β-Actin (internal standard) and TIMP-1 mRNA levels were determined by real-time reverse transcriptase PCR (RT-PCR) using the TaqMan^®^ RNA-to-CT^™^ 1-Step Kit and TaqMan^®^ Gene Expression Assays (Applied Biosystems, Darmstadt, Germany) according to the manufacturer’s instructions.

### Statistics

Comparisons among groups were carried out with one-way ANOVA followed by *post-hoc* Bonferroni or Dunnett tests. In Figure 5, evaluation of statistical significance was confined to the respective groups of interest: vehicle controls (first triplet) versus cisplatin-treated cells (second triplet) and groups of the second triplet versus cisplatin-treated cells in the presence of TIMP-1 siRNA (third triplet). IC_50_ values were calculated by nonlinear regression of log(inhibitor) vs. response using least squares as the fitting method in a 4-parameter calculation with a variable slope. Concentrations (X) were transformed into log(X). Nonlinear regression was calculated using the formula: Y = Bottom + (Top-Bottom)/(1+10^((LogIC50-X)^*^Hill Slope)). Bottom and top are plateaus of minimal (defined as 100% viability) or maximal (defined as 0% viability) loss of viability in response to the concentrations (X). IC_50_ represents loss of viability halfway between the bottom and top. All statistical analyses were performed using GraphPad Prism 5.00 (GraphPad Software, San Diego, CA).

## Abbreviations

Cisplatin: cis-diamminedichloroplatinum(II)
CM: conditioned media
DMEM: Dulbecco´s modified Eagle´s medium
DMF: N,N-dimethylformamide
DMSO: dimethyl sulfoxide
EDTA: ethylenediaminetetraacetic acid
EGFR: epidermal growth factor receptor
FCS: fetal calf serum
HEPES: 4-(2-hydroxyethyl)-1-piperazineethanesulfonic acid
HUVECs: human umbilical vein endothelial cells
LDM: low-dose metronomic
MMP: matrix metalloproteinase
NSCLC: non-small cell lung cancer
PBS: phosphate-buffered saline
PD98059: 2-(2-amino-3-methoxyphenyl)-4H-1-benzopyran-4-one
SB203580: 4-(4-fluorophenyl)-2-(4-methylsulfinylphe-nyl)-5-(4-pyridyl)imidazole
siRNA: small interfering RNA
TIMP-1: tissue inhibitor of matrix metalloproteinases-1
UCM: unconditioned media
WST-1: 4-[3-(4-iodophenyl)-2-(4-nitrophenyl)-2H-5-tetrazolio]-1.3-benzene disulfonate

## References

[1] Folkman J (1972). Anti-angiogenesis: new concept for therapy of solid tumors. Ann Surg.

[2] Ferrara N, Gerber HP, LeCouter J (2003). The biology of VEGF and its receptors. Nat Med.

[3] Iacovelli R, Alesini D, Palazzo A, Trenta P, Santoni M, De Marchis L, Cascinu S, Naso G, Cortesi E (2014). Targeted therapies and complete responses in first line treatment of metastatic renal cell carcinoma. A meta-analysis of published trials. Cancer Treat Rev.

[4] Herbst RS, Heymach JV, Lippman SM (2008). Lung cancer. N Engl J Med.

[5] Reck M, Heigener DF, Mok T, Soria JC, Rabe KF (2013). Management of non-small-cell lung cancer: recent developments. Lancet.

[6] Mok T, Yang JJ, Lam KC (2013). Treating patients with EGFR-sensitizing mutations: first line or second line—is there a difference?. J Clin Oncol.

[7] Muscella A, Vetrugno C, Biagioni F, Calabriso N, Calierno MT, Fornai F, De Pascali SA, Marsigliante S, Fanizzi FP (2016). Antitumour and antiangiogenic activities of [Pt(O,O′-acac)(γ-acac)(DMS)] in a xenograft model of human renal cell carcinoma. Br J Pharmacol.

[8] Li W, Wan L, Zhai LY, Wang J (2014). Effects of SC-560 in combination with cisplatin or taxol on angiogenesis in human ovarian cancer xenografts. Int J Mol Sci.

[9] Hijaz M, Das S, Mert I, Gupta A, Al-Wahab Z, Tebbe C, Dar S, Chhina J, Giri S, Munkarah A, Seal S, Rattan R (2016). Folic acid tagged nanoceria as a novel therapeutic agent in ovarian cancer. BMC Cancer.

[10] Liu P, Gou M, Yi T, Qi X, Xie C, Zhou S, Deng H, Wei Y, Zhao X (2012). The enhanced antitumor effects of biodegradable cationic heparin-polyethyleneimine nanogels delivering HSulf-1 gene combined with cisplatin on ovarian cancer. Int J Oncol.

[11] Rattan R, Graham RP, Maguire JL, Giri S, Shridhar V (2011). Metformin suppresses ovarian cancer growth and metastasis with enhancement of cisplatin cytotoxicity *in vivo*. Neoplasia.

[12] Li DN, Wang L, Wang L, Li S, Wang YB (2016). Expression of Inhibitor of Differentiation-1 and its effects on angiogenesis in gastric cancer. Cancer Biother Radiopharm.

[13] Lee JG, Wu R (2015). Erlotinib-cisplatin combination inhibits growth and angiogenesis through c-MYC and HIF-1α in EGFR-mutated lung cancer *in vitro* and *in vivo*. Neoplasia.

[14] Ma YP, Yang Y, Zhang S, Chen X, Zhang N, Wang W, Cao ZX, Jiang Y, Zhao X, Wei YQ, Deng HX (2010). Efficient inhibition of lung cancer in murine model by plasmid-encoding VEGF short hairpin RNA in combination with low-dose DDP. J Exp Clin Cancer Res.

[15] Jiang QQ, Fan LY, Yang GL, Guo WH, Hou WL, Chen LJ, Wei YQ (2008). Improved therapeutic effectiveness by combining liposomal honokiol with cisplatin in lung cancer model. BMC Cancer.

[16] Lien K, Georgsdottir S, Sivanathan L, Chan K, Emmenegger U (2013). Low-dose metronomic chemotherapy: a systematic literature analysis. Eur J Cancer.

[17] Pasquier E, Kavallaris M, André N (2010). Metronomic chemotherapy: new rationale for new directions. Nat Rev Clin Oncol.

[18] Nakata B, Mitachi Y, Tsuji A, Yamamitsu S, Hirata K, Shirasaka T, Hirakawa K (2004). Combination phase I trial of a novel oral fluorouracil derivative S-1 with low-dose cisplatin for unresectable and recurrent gastric cancer (JFMC27-9902). Clin Cancer Res.

[19] Nakata B, Tsuji A, Mitachi Y, Taenaka N, Kamano T, Oikawa K, Onoda N, Kambe M, Takahashi M, Shirasaka T, Morita S, Sakamoto J, Tanaka Y (2010). Phase II trial of S-1 plus low-dose cisplatin for unresectable and recurrent gastric cancer (JFMC27-9902 Step2). Oncology.

[20] Himmelstein KJ, Patton TF, Belt RJ, Taylor S, Repta AJ, Sternson LA (1981). Clinical kinetics on intact cisplatin and some related species. Clin Pharmacol Ther.

[21] Shen FZ, Wang J, Liang J, Mu K, Hou JY, Wang YT (2010). Low-dose metronomic chemotherapy with cisplatin: can it suppress angiogenesis in H22 hepatocarcinoma cells?. Int J Exp Pathol.

[22] Adhim Z, Lin X, Huang W, Morishita N, Nakamura T, Yasui H, Otsuki N, Shigemura K, Fujisawa M, Nibu K, Shirakawa T (2012). E10A, an adenovirus-carrying endostatin gene, dramatically increased the tumor drug concentration of metronomic chemotherapy with low-dose cisplatin in a xenograft mouse model for head and neck squamous-cell carcinoma. Cancer Gene Ther.

[23] Jian W, Levitt JM, Lerner SP, Sonpavde G (2009). Preclinical antitumor and antiangiogenic activity of a metronomic schedule of cisplatin against human transitional cell carcinoma (TCC). J Clin Oncol.

[24] Bijman MN, van Nieuw Amerongen GP, Laurens N, van Hinsbergh VW, Boven E (2006). Microtubule-targeting agents inhibit angiogenesis at subtoxic concentrations, a process associated with inhibition of Rac1 and Cdc42 activity and changes in the endothelial cytoskeleton. Mol Cancer Ther.

[25] Ramer R, Fischer S, Haustein M, Manda K, Hinz B (2014). Cannabinoids inhibit angiogenic capacities of endothelial cells via release of tissue inhibitor of matrix metalloproteinases-1 from lung cancer cells. Biochem Pharmacol.

[26] Ramer R, Eichele K, Hinz B (2007). Upregulation of tissue inhibitor of matrix metalloproteinases-1 confers the anti-invasive action of cisplatin on human cancer cells. Oncogene.

[27] Gong M, Yu B, Wang J, Wang Y, Liu M, Paul C, Millard RW, Xiao DS, Ashraf M, Xu M (2017). Mesenchymal stem cells release exosomes that transfer miRNAs to endothelial cells and promote angiogenesis. Oncotarget.

[28] Reddel RR, Ke Y, Gerwin BI, McMenamin MG, Lechner JF, Su RT, Brash DE, Park JB, Rhim JS, Harris CC (1988). Transformation of human bronchial epithelial cells by infection with SV40 or adenovirus-12 SV40 hybrid virus, or transfection via strontium phosphate coprecipitation with a plasmid containing SV40 early region genes. Cancer Res.

[29] Amstad P, Reddel RR, Pfeifer A, Malan-Shibley L, Mark GE, Harris CC (1988). Neoplastic transformation of a human bronchial epithelial cell line by a recombinant retrovirus encoding viral Harvey ras. Mol Carcinog.

[30] Wan YW, Raese RA, Fortney JE, Xiao C, Luo D, Cavendish J, Gibson LF, Castranova V, Qian Y, Guo NL (2012). A smoking-associated 7-gene signature for lung cancer diagnosis and prognosis. Int J Oncol.

[31] Yang L, Moghaddas S, Dezvareh H, Belkacemi L, Bark SJ, Bose RN, Do LH (2016). Insights into the anti-angiogenic properties of phosphaplatins. J Inorg Biochem.

[32] Montiel M, Urso L, de la Blanca EP, Marsigliante S, Jiménez E (2009). Cisplatin reduces endothelial cell migration via regulation of type 2-matrix metalloproteinase activity. Cell Physiol Biochem.

[33] Eastman A (2017). Improving anticancer drug development begins with cell culture: misinformation perpetrated by the misuse of cytotoxicity assays. Oncotarget.

[34] Kumar P, Benedict R, Urzua F, Fischbach C, Mooney D, Polverini P (2005). Combination treatment significantly enhances the efficacy of antitumor therapy by preferentially targeting angiogenesis. Lab Invest.

[35] Moses MA, Sudhalter J, Langer R (1990). Identification of an inhibitor of neovascularization from cartilage. Science.

[36] Johnson MD, Kim HR, Chesler L, Tsao-Wu G, Bouck N, Polverini PJ (1994). Inhibition of angiogenesis by tissue inhibitor of metalloproteinase. J Cell Physiol.

[37] Seandel M, Noack-Kunnmann K, Zhu D, Aimes RT, Quigley JP (2001). Growth factor-induced angiogenesis *in vivo* requires specific cleavage of fibrillar type I collagen. Blood.

[38] Martin DC, Sanchez-Sweatman OH, Ho AT, Inderdeo DS, Tsao MS, Khokha R (1999). Transgenic TIMP-1 inhibits simian virus 40 T antigen-induced hepatocarcinogenesis by impairment of hepatocellular proliferation and tumor angiogenesis. Lab Invest.

[39] Guedez L, McMarlin AJ, Kingma DW, Bennett TA, Stetler-Stevenson M, Stetler-Stevenson WG (2001). Tissue inhibitor of metalloproteinase-1 alters the tumorigenicity of Burkitt’s lymphoma via divergent effects on tumor growth and angiogenesis. Am J Pathol.

[40] Ikenaka Y, Yoshiji H, Kuriyama S, Yoshii J, Noguchi R, Tsujinoue H, Yanase K, Namisaki T, Imazu H, Masaki T, Fukui H (2003). Tissue inhibitor of metalloproteinases-1 (TIMP-1) inhibits tumor growth and angiogenesis in the TIMP-1 transgenic mouse model. Int J Cancer.

[41] Avalos BR, Kaufman SE, Tomonaga M, Williams RE, Golde DW, Gasson JC (1988). K562 cells produce and respond to human erythroid-potentiating activity. Blood.

[42] Chesler L, Golde DW, Bersch N, Johnson MD (1995). Metalloproteinase inhibition and erythroid potentiation are independent activities of tissue inhibitor of metalloproteinases-1. Blood.

[43] Jung KK, Liu XW, Chirco R, Fridman R, Kim HR (2006). Identification of CD63 as a tissue inhibitor of metalloproteinase-1 interacting cell surface protein. EMBO J.

[44] Chirco R, Liu XW, Jung KK, Kim HR (2006). Novel functions of TIMPs in cell signaling. Cancer Metastasis Rev.

[45] Sancéau J, Truchet S, Bauvois B (2003). Matrix metalloproteinase-9 silencing by RNA interference triggers the migratory-adhesive switch in Ewing’s sarcoma cells. J Biol Chem.

[46] Zeng H, Briske-Anderson M (2005). Prolonged butyrate treatment inhibits the migration and invasion potential of HT1080 tumor cells. J Nutr.

[47] Cheung LW, Leung PC, Wong AS (2006). Gonadotropin-releasing hormone promotes ovarian cancer cell invasiveness through c-Jun NH2-terminal kinase-mediated activation of matrix metalloproteinase (MMP)-2 and MMP-9. Cancer Res.

[48] Ren T, Shan J, Li M, Qing Y, Qian C, Wang G, Li Q, Lu G, Li C, Peng Y, Luo H, Zhang S, Yang Y (2015). Small-molecule BH3 mimetic and pan-Bcl-2 inhibitor AT-101 enhances the antitumor efficacy of cisplatin through inhibition of APE1 repair and redox activity in non-small-cell lung cancer. Drug Des Devel Ther.

[49] Chintala SK, Ali-Osman F, Mohanam S, Rayford A, Go Y, Gokaslan ZL, Gagercas E, Venkaiah B, Sawaya R, Nicolson GL, Rao JS (1997). Effect of cisplatin and BCNU on MMP-2 levels in human glioblastoma cell lines *in vitro*. Clin Exp Metastasis.

[50] Geng Y, Wang J, Jing H, Wang HW, Bao YX (2014). Inhibitory effect of dexamethasone on Lewis mice lung cancer cells. Genet Mol Res.

[51] Tas F, Duranyildiz D, Soydinc HO, Cicin I, Selam M, Uygun K, Disci R, Yasasever V, Topuz E (2008). Effect of maximum-tolerated doses and low-dose metronomic chemotherapy on serum vascular endothelial growth factor and thrombospondin-1 levels in patients with advanced nonsmall cell lung cancer. Cancer Chemother Pharmacol.

[52] Coxon A, Ziegler B, Kaufman S, Xu M, Wang H, Weishuhn D, Schmidt J, Sweet H, Starnes C, Saffran D, Polverino A (2012). Antitumor activity of motesanib alone and in combination with cisplatin or docetaxel in multiple human non-small-cell lung cancer xenograft models. Mol Cancer.

[53] Kong C, Zhu Y, Sun C, Li Z, Sun Z, Zhang X, Takanaka I (2005). Inhibition of tumor angiogenesis during cisplatin chemotherapy for bladder cancer improves treatment outcome. Urology.

[54] Kerbel RS, Kamen BA (2004). The anti-angiogenic basis of metronomic chemotherapy. Nat Rev Cancer.

[55] Munoz R, Shaked Y, Bertolini F, Emmenegger U, Man S, Kerbel RS (2005). Anti-angiogenic treatment of breast cancer using metronomic low-dose chemotherapy. Breast.

[56] Grant DS, Tashiro K, Segui-Real B, Yamada Y, Martin GR, Kleinman HK (1989). Two different laminin domains mediate the differentiation of human endothelial cells into capillary-like structures *in vitro*. Cell.

[57] Hinz B, Rösch S, Ramer R, Tamm ER, Brune K (2005). Latanoprost induces matrix metalloproteinase-1 expression in human nonpigmented ciliary epithelial cells through a cyclooxygenase-2-dependent mechanism. FASEB J.

